# Five cases of tonsillectomy and steroid pulse therapy for recurrent immunoglobulin A nephropathy after kidney transplantation

**DOI:** 10.1007/s13730-013-0098-6

**Published:** 2013-09-12

**Authors:** Yoshie Hoshino, Yasutomo Abe, Mariko Endo, Sachiko Wakai, Hiroki Shirakawa, Osamu Hotta, Hideki Ishida, Kazunari Tanabe, Ken Tsuchiya, Kosaku Nitta

**Affiliations:** 1 Department of Medicine, Kidney Center, Tokyo Women’s Medical University, 8-1 Kawada-cho, Shinjuku-ku, Tokyo 162-8666 Japan; 2 Department of Nephrology, Okubo Hospital, Tokyo Metropolitan Health and Medical Treatment Corporation, Tokyo, Japan; 3 Department of Transplantation, Okubo Hospital, Tokyo Metropolitan Health and Medical Treatment Corporation, Tokyo, Japan; 4 Hotta Osamu Clinic, Miyagi, Japan; 5 Department of Urology, Tokyo Women’s Medical University, Tokyo, Japan

**Keywords:** Recurrent IgA nephropathy, Tonsillectomy, Steroid pulse, Kidney transplantation

## Abstract

Five cases of recurrent immunoglobulin A nephropathy (IgAN) after kidney transplantation were successfully treated by tonsillectomy and steroid pulse therapy (SPT). The clinical background and pathology in the five cases were different, but good results were obtained in all of them. In cases 1 and 2, mild recurrent IgAN developed and failed to remit after tonsillectomy alone, but a remission was achieved in both cases after SPT. In case 3, highly active recurrent IgAN with crescent lesions developed 13 years after kidney transplantation, and a remission was achieved after SPT. In case 4, renal biopsy specimens showed pathological findings of recurrent IgAN with tubulitis, and hematuria and proteinuria resolved after SPT. In case 5, the biopsy findings indicated recurrent IgAN with chronic rejection. Tonsillectomy was followed by resolution of the proteinuria, and a remission was achieved after SPT. In conclusion, SPT is effective in inducing a remission of recurrent IgAN when tonsillectomy alone fails.

## Introduction

Recurrent IgA nephropathy (re-IgAN) after kidney transplantation (KT) influences graft survival but there is no established standard treatment. We report five cases of re-IgAN with different characteristics, which achieved remission by tonsillectomy and steroid pulse therapy (SPT).

## Case reports

The clinical characteristics of the five cases are shown in Table 1. All cases were confirmed a biopsy-proven IgAN before entering dialysis in each hospital.

**Table 1:** Clinical profiles of the cases

	Case 1	Case 2	Case 3	Case 4	Case 5
Gender	M	M	M	M	M
Age at onset (years)	10	Approximately 28	Approximately 15	20	12
Onset to dialysis (years)	14	Approximately 5	Approximately 18	10	20
Dialysis period (years)	8	5	2	3	3
Immunosuppressant	FK MMF MP Basiliximab	FK MF MP Basiliximab	FK MZ → MMF MP Basiliximab	CyA MMF MP Basiliximab Ritu DFPP × 3	FK MF MP Basiliximab Ritu
Age at transplant (years)	32	38	35	33	35
ABO compatible	Compatible	Compatible	Compatible	Incompatible	Compatible
Anti-HLA antibody	–	–	–	–	+
Crossmatch (CDC, FCXM)	–	–	–	–	–
Recipient	Mother	Brother	Mother	Mother	Mother
Age at recurrence (years)	37	41	48	36	36
Urinary findings at recurrence	Prot− RBC1+	Prot− RBC2+	Prot2+ RBC2+	Prot1+ RBC2+	Prot1+ RBC1+
SCr (mg/dl) at recurrence	1.48	1	1.46	1.18	1.44
Age at tonsillectomy (years)	37	43	50	36	36
Courses of steroid pulse	mPSL500 mg/day for 3 days 1 course	mPSL500 mg/day for 3 days 2 courses	mPSL500 mg/day for 3 days 3 courses	mPSL500 mg/day for 2 days 1 course	mPSL500 mg/day for 3 days 1 course
Latent urinary findings	Prot− RBC±	Prot− RBC−	Prot− RBC−	Prot− RBC1+	Prot− RBC−
Latent SCr (mg/dl)	1.62	0.96	1.58	1.18	1.8
Renal pathology at IgAN diagnosis	*H-grade I A/C	H-grade I A/C	Focal proliferative glomerulonephritis with crescents, H-grade II A/C	H-grade I A/C, IF/TA moderate	H-grade II A/C, chronic active T cell mediated rejection

*CDC* complement-dependent cytotoxicity, *FCXM* flow cytometry crossmatch, *Tac* tacrolimus, *CyA* cyclosporin *MMF* mycophenolate mofetil, *MZ* mizoribine, *Ritu* rituximab, *MP* methylprednisolone, *mPSL* methylprednisolone, *DFPP* double filtration plasmapheresis

* Histological grade based on “IgA nephropathy practice guideline, third edition” [13]

### Case 1

The patient was performed ABO-compatible KT at 32 years of age. IgA deposition without C3 was identified by 0-h biopsy, but no active lesion was detected and it decreased by the second-year biopsy. Hematuria appeared in the fifth year, and mild re-IgAN with mesangial proliferation was diagnosed. Despite tonsillectomy, abnormal urinary findings did not resolve. The addition of SPT achieved remission.

### Case 2

The patient was performed ABO-compatible KT at 38 years of age. The 0-h biopsy specimen revealed little IgA deposition without C3 deposition nor active lesion. It remained by the 2-week biopsy specimen, but it then decreased and urinary findings were normal. Hematuria developed in the third-year and the fifth-year biopsy revealed re-IgAN with C3 deposition and mild proliferative change. The patient underwent tonsillectomy and SPT but hematuria persisted. Two months later, SPT was administered a second time and remission resulted. The seventh-year biopsy showed no active lesion.

### Case 3

**Fig. 1:**
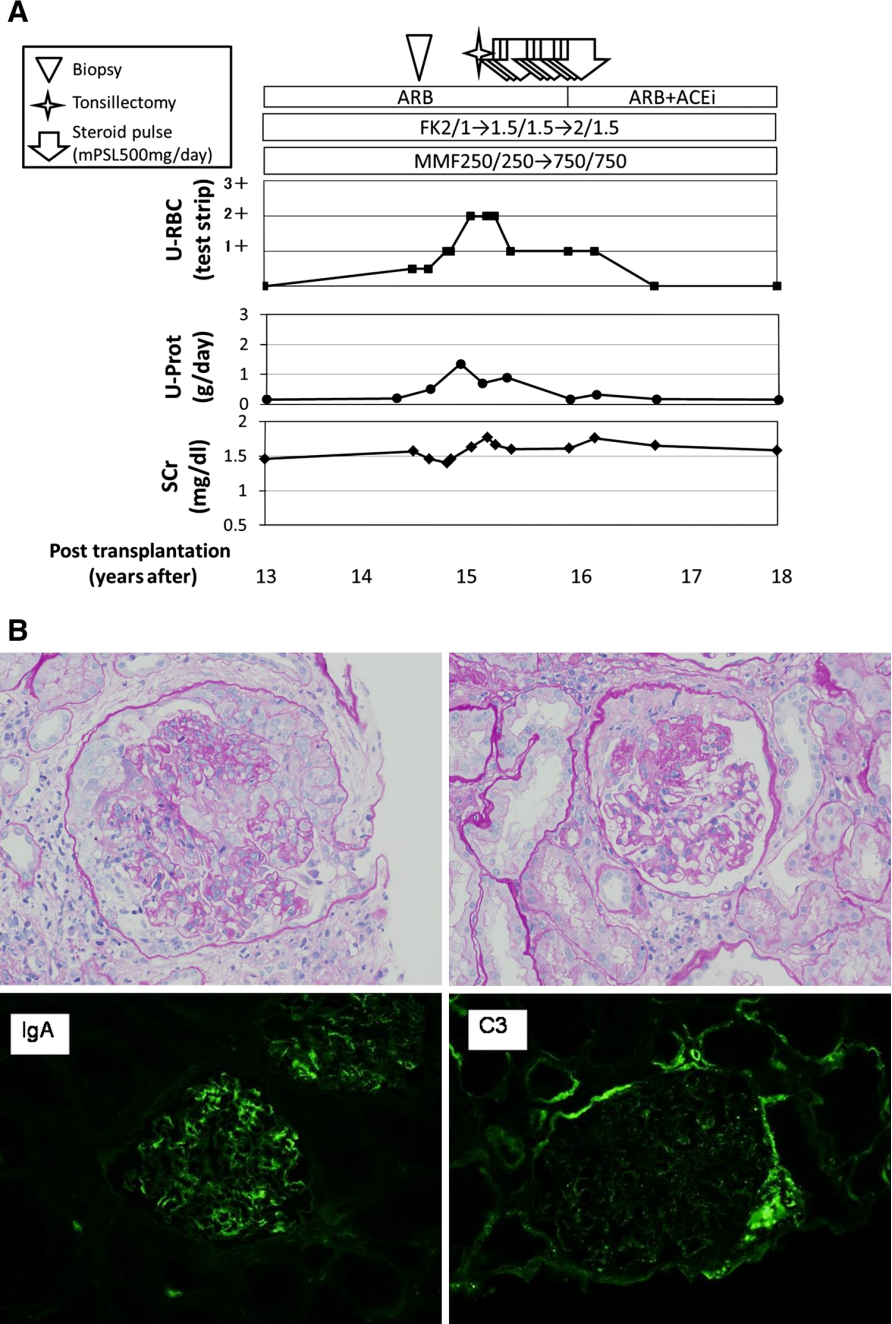
Clinical course and pathological changes at re-IgAN diagnosis in case 3: proteinuria and hematuria developed and the 14th-year biopsy revealed high-activity re-IgAN with crescentic lesions (**b**). After 1 year of tonsillectomy and SPT, he achieved clinical remission (**a**)

The patient was performed ABO-compatible KT at 35 years of age. Insulin therapy was initiated because of steroidal diabetes. Proteinuria developed in the 13th year, and immunosuppressive drugs were switched. But proteinuria worsened and hematuria developed. Biopsy revealed high-activity re-IgAN with cellular crescents and fibrocellular crescentic lesions (Fig. 1). After tonsillectomy and SPT, remission was achieved. The insulin dose was increased only during SPT.

### Case 4

The patient was performed ABO-incompatible KT at 33 years of age. Two years later, hematuria (2+ to 3+) developed. Despite increasing the oral steroids and performing tonsillectomy, hematuria failed to improve and proteinuria developed. Biopsy revealed re-IgAN and clearly demarcated inflammatory cell infusion of medullary rays. After SPT, proteinuria resolved and hematuria improved (1+).

### Case 5

The patient was performed ABO-compatible KT at 35 years of age. The 0-h biopsy showed normal findings. Proteinuria and hematuria developed 6 months later, and the first-year biopsy revealed mesangial-area expansion and IgA with C3 deposition. After tonsillectomy, proteinuria and hematuria disappeared and IgA deposition improved. In the third year, hematuria developed again and serum creatinine (SCr) worsened. Biopsy showed renewed IgA deposition with T cell-mediated rejection. After SPT, SCr and IgA deposition improved and rejection resolved following biopsy 2 months later. The fourth-year biopsy showed no active lesions.

## Discussion

We performed tonsillectomy and SPT in five cases of re-IgAN; all of them showed a good result, without side effects. Cases 1 and 2 showed mild activity. Both cases showed IgA deposition at the 0-h biopsy, but this decreased at the next biopsy. Generally, transmitted IgA deposition from the donor disappears within 3 years; their re-IgA were not derived from the donor. Tonsillectomy alone failed to achieve remission; adding one course of SPT in case 1 and two courses in case 2 resulted in a remission. Severe activity in case 3 achieved remission after tonsillectomy and 3 courses of SPT. Both re-IgAN and lymphocytic infusion were observed in case 4, but a clearly demarcated area of infiltration may have represented tubulointerstitial disorder due to urinary stasis. In case 5, chronic rejection caused SCr to increase. However, since tubulointerstitial disorders and rejection are not associated with hematuria, it occurs by re-IgAN. Tonsillectomy and SPT improved clinical findings in both cases, as steroids are expected to improve all of these disorders.

re-IgAN is diagnosed by biopsy in 25–60 % of KT recipients with a history of primary IgAN (p-IgAN) [1–4]. Of these, graft failure develops in 18.7–26.5 % and graft loss occurs in 1.3–10 % 10 years later [3, 5, 6]. re-IgAN influences long-term graft outcome [4, 7].

No standard treatment for re-IgAN has been established. It is concerned that SPT is over-immunosuppression in recipients. Sakai et al. [8] reported cases of re-IgAN remission by tonsillectomy alone. Kennoki et al. [9] compared tonsillectomy and control groups and reported a significant decrease in proteinuria in the tonsillectomy group 6 months later. Clayton et al. [10] reported that steroid therapy 2 years after KT decreased the risk of graft loss, and Tsuchiya et al. [11] reported two cases of re-IgAN remission by tonsillectomy and SPT.

Tonsillectomy was performed in 21 cases of re-IgAN at our hospital during the period 2008–2012. A completed remission was achieved in 12 of the 18 cases with abnormal urinary findings by tonsillectomy alone. SPT was administered in five of six latter cases and complete remission was achieved in four. These results suggest that, in some cases, remission can be achieved by tonsillectomy alone, whereas combination with SPT and tonsillectomy is required in other cases. In addition, the numbers of courses and steroid dosage required to achieve remission differs in each case. Hotta et al. [12] reported that remission rates decreased as the clinical course duration increased. If treatment is initiated early in recurrence and activity is mild, remission may be achieved by tonsillectomy alone. However, high-activity cases may require strong immunosuppressive therapy with SPT. P-IgAN occurs in youths and they undergo transplantation during youth. In our hospital, re-IgAN patients underwent transplantation at an average age of 36 years, with an approximately 5 years interval to recurrence. They needed the treatment in order to improve long-term graft survival.

There are some issues to add to the investigation. First, these five cases of re-IgAN were identified by the episode biopsy and not only IgAN findings but also other findings, such as chronic rejection and tubulointerstitial disorder. We cannot exclude the effect of these comorbidities. Further study is required for the cases of re-IgAN alone. Second, the protocol of tonsillectomy and SPT, and third the long-term outcome of these cases. Future study is necessary.

In conclusion, SPT is effective in achieving clinical remission in re-IgAN where tonsillectomy alone fails, and SPT does not increase the complication rate.
